# Neighborhood-scale lead (Pb) speciation in Akron, Ohio (USA) soils: primary sources, post-deposition diagenesis, and high concentrations of labile Pb

**DOI:** 10.1007/s10653-024-01954-z

**Published:** 2024-04-09

**Authors:** Nicholas Santoro, David M. Singer, Bridget K. Mulvey, Katrina Halasa, Nadya Teutsch, Allie Shedleski, Madison Wood

**Affiliations:** 1 Department of Earth Sciences, Kent State University, Kent, OH 44242 USA; 2 School of Teaching, Learning and Curriculum Studies, Kent State University, Kent, OH 44242 USA; 3 Akron Public Schools, Akron, OH 44308 USA; 4 Geochemistry and Environmental Geology Division, Geological Survey of Israel, 9692100 Jerusalem, Israel

**Keywords:** Urban soil, Lead, Metal contamination, Pb isotopes

## Abstract

Lead (Pb) poses a significant risk to infants and children through exposure to contaminated soil and dust. However, there is a lack of information on Pb speciation and distribution at the neighborhood-scale. This work aimed to determine: (1) the distribution of acid-extractable (labile) Pb and other metals ([M]_AE_) in two neighborhoods in Akron, Ohio (USA) (Summit Lake and West Akron; n = 82 samples); and (2) Pb speciation and potential sources. Total metal concentration ([M]_T_) and [M]_AE_ was strongly correlated for Pb and Zn (R^2^ of 0.66 and 0.55, respectively), corresponding to 35% and 33% acid-extractability. Lead and Zn exhibited a strong positive correlation with each other (R^2^ = 0.56 for M_T_ and 0.68 for M_AE_). Three types of Pb-bearing phases were observed by electron microscopy: (1) galena (PbS)-like (5–10 μm); (2) paint chip residuals (10–20 μm); and (3) Pb-bearing Fe-oxides (20 μm). Isotope ratio values for Pb_AE_ were 1.159 to 1.245 for ^206^Pb/^207^Pb, and 1.999 to 2.098 for ^208^Pb/^206^Pb, and there was a statistically significant difference between the two neighborhoods (*p* = 0.010 for ^206^Pb/^207^Pb and *p* = 0.009 for ^208^Pb/^206^Pb). Paint and petrol are the dominant sources of Pb, with some from coal and fly ash. Lead speciation and distribution is variable and reflects a complex relationship between the input of primary sources and post-deposition transformations. This work highlights the importance of community science collaborations to expand the reach of soil sampling and establish areas most at risk based on neighborhood-dependent Pb speciation and distribution for targeted remediation.

**Supplementary Information:**

The online version contains supplementary material available at 10.1007/s10653-024-01954-z.

## Introduction

Lead (Pb), a highly toxic element, has been introduced into the environment through a variety of anthropogenic activities. In United States (US) urban environments, the primary sources of Pb are from historic leaded paint and petrol use. Lead has been added to paint for centuries to improve pigment durability and color with commercial and industrial paints containing up to 15% Pb (Filippelli et al., 2015). Housing booms in the United States throughout the early and mid-1900s resulted in upwards of 24 million new homes being painted with Pb-based paints (Filippelli et al., 2015). It is estimated that 31 million pre-1978 houses still contain Pb-based paint, including 3.8 million of them that have one or more children under the age of six living there (USEPA, 2023). Lead can be released to the environment via aerosolized particles during renovation, paint chipping off of high-friction surfaces (window and door frames), and peeling paint flaking off into adjacent soils (Schuch et al., 2017). Beginning in 1921, gasoline manufactures introduced Pb additives for commercial vehicle use to help reduce engine knocking, which lasted until 1996 in the US, when leaded gasoline was finally phased out (Schwarz et al., 2012). During peak leaded gasoline use in the 1970s, as much as 1.2 g Pb L^−1^ were added to gasoline (Newell & Rogers, 2003) with up to five million tons of Pb released to the atmosphere (Nriagu, 1999), which resulted in an estimated accumulation of Pb in soils of 10 mg Pb m^−2^ (Chow & Johnstone, 1965; Hwang et al., 2016). The leaded gasoline ban resulted in US emission rate decreases from 12,300 µg km^−1^ in 1977 (Pierson & Brachaczek, 1982) to 0.156 to 0.797 μg km^−1^ in 2003 (Geller et al., 2006). Other sources of Pb, and other commonly occurring metal contaminants including copper (Cu) and zinc (Zn) in surface soils and sediments in urban and industrial environments include deposition of dust and aerosol particles from smelting activities, corrosion of metal structures (e.g., galvanized roofs and fences), contamination from former and current industrial, agricultural, or horticultural use of the land, and bonfires and accidental fires (Alloway, 2013).

The summation of these sources can result in elevated Pb exposure pathways that carry the greatest risk for infants and children due to their smaller size and proportionately larger dose of ingested toxins, their proximity to ground soil and indoor dust, and their oral exploratory and pica behaviors (Hauptman et al., 2017; Lanphear et al., 1998). Exposure to Pb, even at low-levels, is a causal risk factor for diminished intellectual and academic abilities, higher rates of neurobehavioral disorders such as hyperactivity and attention deficits, and lower birth weight in children (Mielke, 1999). Further, it is likely that Pb exposure-related health effects occur at lower blood Pb levels (BLL) than previously thought (NTP, 2012). There is no identified BLL without deleterious health effects in children (CDC, 2012; Gilbert & Weiss, 2006), and no effective treatments ameliorate the permanent developmental effects of Pb toxicity (Flora et al., 2012). Following the peak in Pb use in the United States, BLLs in children between ages 1 to 5 was as high as 15 µg dL^−1^ (Egan et al., 2021). Despite the phase-out of Pb from common industrial sources, over half a million children in the United States were reported to have BLL values above 3.5 µg dL^−1^ in 2012 and more than half of all children in the country have a BLL above 1 µg dL^−1^ (Hauptman et al., 2017). However, the risk of exposure is not equally distributed among the population, with black children making up more than 50% of children with BLL greater than 1 µg dL^−1^ (Yeter et al., 2020), underscoring the use of high BLLs as a socioeconomic indicator of inequality.

Ingestion and/or inhalation of Pb from soil and dust are potentially significant exposure pathways that can contribute up to approximately 70% of a child’s BLL (Laidlaw et al., 2017; Mielke et al., 2017). Although reducing Pb exposure from residential and industrial Pb hazards is an effective way to prevent or control childhood Pb exposure (Lanphear et al., 2002), Pb uptake mechanism(s) typically focus on in-door exposure to Pb-bearing drinking water and/or paint (Schnur & John, 2014; Triantafyllidou & Edwards, 2012). Therefore, the importance of outdoor Pb exposure from soil and soil-derived airborne particles (i.e., aerosols and road dust) has likely been overlooked. Recent work in the US has highlighted that exposure to paint alone cannot account for elevated BLL patterns in children due to the following set of observations: (1) Elevated BLLs in neighborhoods with limited to no Pb-paint use, such as inner-city Baltimore, Maryland, was found to have high Pb concentrations in soils (Mielke, 1999; Mielke et al., 1983); (2) Seasonable BLL patterns of summer highs and winter lows suggesting a source external to homes; if exposure was primarily from in-house Pb-paint and household dust, BLL would be highest during the winter when children spend more time indoors (Laidlaw et al., 2016); (3) Decreases in BLL in children have been observed when contaminated soils are covered and/or remediated, most dramatically observed in New Orleans, Louisiana in the decade following Hurricane Katrina in 2005, when uncontaminated Gulf of Mexico sediments covered contaminated soils (Mielke et al., 2017); (4) Re-suspension of Pb-contaminated soils from vehicle emissions of leaded petrol use has also been shown to be a dominant source of atmospheric Pb across a range of urban and rural environments and correlated with high exposure in children (Laidlaw et al., 2012); and (5) Top soils in general, and urban soils in particular, are the dominant source of airborne Pb in these vicinities, which could explain the continued elevated BLL in urban populations despite a lack of continued Pb emissions from primary sources (Harlavan et al., 2020; Pingitore et al., 2009) due to retention of Pb in soil particles (Markus & McBratney, 2001). These results indicate the importance of Pb in soil and soil-derived airborne particles and its potential as a primary exposure pathway, particularly in urban environments.

Although total Pb concentration in soils can be an indicator of exposure risk, speciation (i.e., its chemical and physical form) exerts a stronger degree of control potential bioaccessibility and toxicity (MacLean et al., 2011b). Speciation of Pb in soils can range from soluble forms such as Pb adsorbed onto organic solids, anglesite (PbSO_4_), lead ammonium sulfate (PbSO_4_·(NH_4_)_2_SO_4_) and hydrocerussite (PbCO_3_·Pb(OH)_2_) to insoluble forms such as metallic lead (Pb^0^), lead tetroxide (Pb_3_O_4_), and monobasic lead sulfate (PbO·PbSO_4_) (Biggins & Harrison, 1980; MacLean et al., 2011b). X-ray spectroscopic and sequential extraction studies have linked Pb speciation in soils and potential bioaccessibility to the solubility of dominant Pb-bearing phases (Bradham et al., 2014; MacLean et al., 2011a; Walker et al., 2011). Speciation is also affected by how Pb is distributed in different particle size fractions and the presence of heterogeneous, physically complex Pb-bearing phases (Ruby et al., 1999; Yan et al., 2017). For example, high Pb concentrations in particles (less than 100 µm) have been linked to historic use of leaded petrol, despite the cessation of those activities several decades prior (Miguel et al., 1997). Formation of Pb-bearing nanoparticles bound within a silica matrix has been observed in contaminated soils down gradient of industrial sources (Schindler & Hochella, 2017). The risk of human exposure to Pb via inhalation and/or ingestion is dependent on the grain size of soil particles or aggregates, particularly for particles less than 50 μm particles (Bi et al., 2013). Further, higher concentrations of total and labile Pb Pb have been found to increase with decreasing particle size in urban soils (Juhasz et al., 2011) and road-deposited sediment (Sutherland, 2003). Labile Pb within contaminated soils is therefore a combination of both chemical speciation (i.e., which Pb-bearing phase(s) are present), and physical speciation (i.e. which particle sizes, mineralogical textures and morphologies Pb is are associated with).

Based on the previous work summarized above, it is apparent that there is a lack of Pb speciation and distribution at the neighborhood-scale, which is critical for establishing the actual risk of exposure from contaminated soils. Although the fraction of labile Pb may be linearly correlated with the total concentration of Pb, the source and/or local speciation may result in a non-linear relationship. The objectives of the current work are: (1) to determine the concentration and distribution of acid-extractable (labile) Pb at the neighborhood-scale, which can be used a proxy for the fraction of potentially bioaccessible Pb (Harvey et al., 2017; Wu et al., 2010); and (2) to determine Pb speciation and potential Pb sources using a suite of techniques including electron microscopy and Pb isotope analyses to explore the relationship between the input of primary sources and post-deposition transformations.

## Methods and materials

### Site description, sample collection, and preparation

**Fig. 1:**
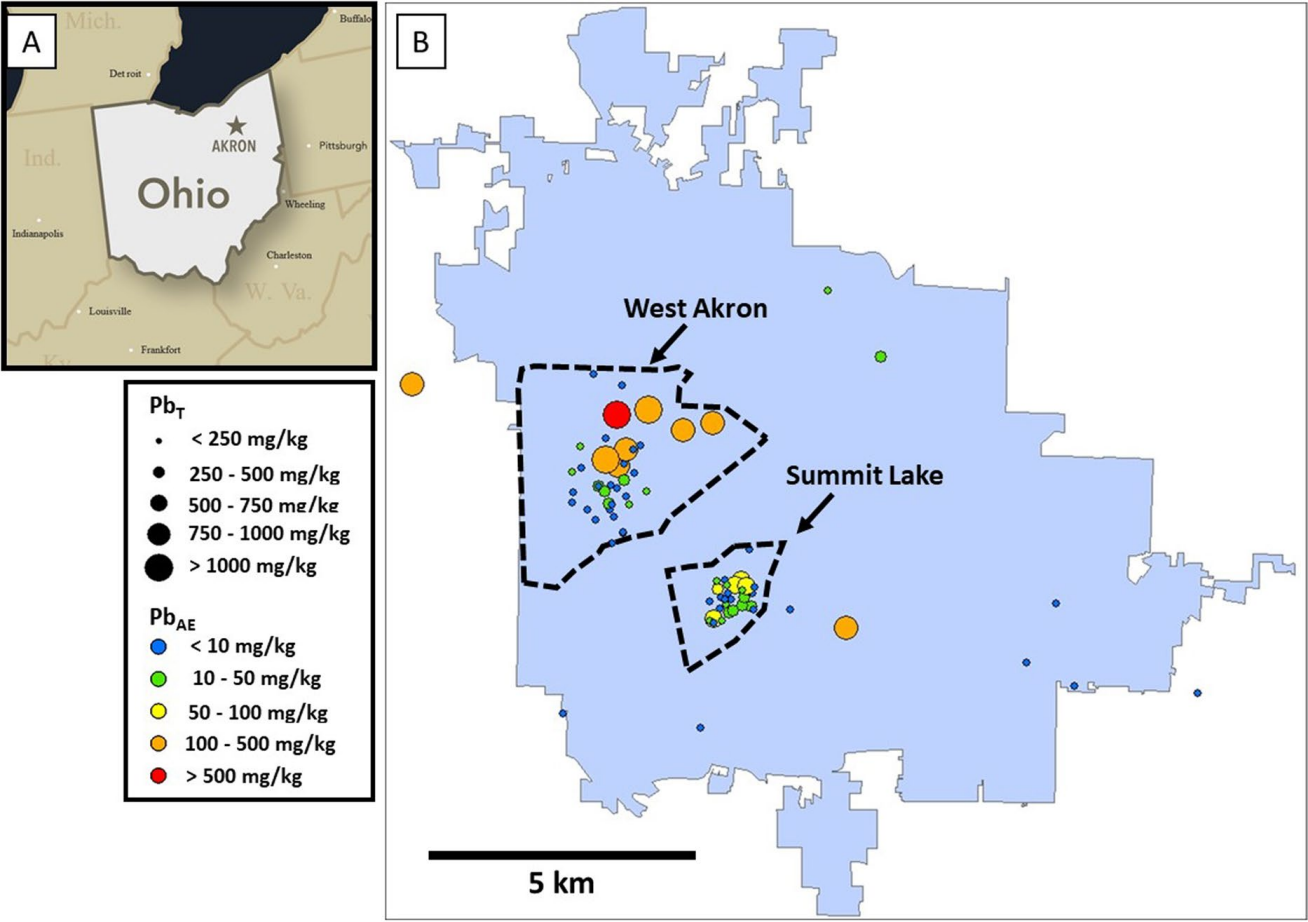
**A** location of Akron, OH (USA); **B** map of Akron (in blue) with the locations of the West Akron and Summit Lake neighborhoods outlined in dashed lines. Each circle represents the location where a soil sample was collected; the symbol size corresponds to the amount of total Pb (Pb_T_) and the symbol color corresponds to the amount of nitric acid-extractable Pb (Pb_AE_)

The study areas were two neighborhoods in Akron, Ohio (USA) (Fig. 1), a metropolitan area with a population of approximately 190,000 (U.S.CensusBureau, 2020). The development of Akron rapidly increased due to the rise of the rubber and tire industries, and population peaked at 290,000 in the 1960s. The two neighborhoods, Summit Lake and West Akron, are dominated by older (pre-1978) housing stock (Schuch et al., 2017). Although BLL data is limited for Akron and Summit County, data from 2012 through 2014 indicated that 4.1% of tested children had elevated BLLs, in comparison to a national average of approximately 2.5% around the same time period (Hauptman et al., 2017), and the distribution of elevated BLL clusters are within the city of Akron forming a ring around the city center including the two neighborhoods that are part of the current study (Schuch et al., 2017). Surface soils samples were collected from the Summit Lake neighborhood in November 2018. To ensure optimal sampling locations and the most efficient sampling route for the Summit Lake samples, initial video analysis of the area was conducted during July 2018. Video cameras and GPS units were mounted on both sides of the vehicle and recorded each house throughout the neighborhood to identify abandoned and unoccupied homes. Each video was then uploaded onto Google Earth and linked with the GPS coordinates from the initial data collection. Analysis of the videos showed a wide distribution of abandoned and unoccupied houses that could serve as sampling locations throughout Summit Lake. Of the large preliminary group of houses, 30 were selected because of their geographically even distribution throughout the neighborhood and because of the presence of nearby road verges (i.e., the strip of grass adjacent to the street and at the edge of a house property) providing a consistent area to sample from. One sample was collected from each block of the roughly grid-like layout of Summit Lake. Samples were collected from the West Akron neighborhood in the spring of 2019 through collaboration with Akron Public Schools (APS), which included 60 middle and high school students from schools on the westside of Akron. The APS students were instructed on how to safely collect and label soil samples from nearby road verges near their homes or nearby locations. Location address and participant addresses were anonymized after sample retrieval. Each sample was collected using trowels and bags supplied by the Kent State Department of Earth Sciences and resulted in the collection of 52 samples primarily from, but not limited to, the West Akron neighborhood. At all locations, a 2 cm deep soil sample was collected after clearing any surface vegetation that may have been present. Each sample was sieved (2 mm) to remove any debris and large rocks. Samples were dried at 40 °C for 12 h using a Binder ED-115 oven and then milled using a SPEX ball mill 8000 M with tungsten carbide balls for 10 min to produce homogenous powder. The milling canister was cleaned using isopropyl alcohol between runs to prevent cross contamination. Milled soils samples were used for solid phase characterization and acid extractions (described below) and sieved-only samples were analyzed by electron microscopy.

### Solid phase characterization

Loss on ignition (LOI) was used as a proxy for organic matter content (Dean, 1974; Konen et al., 2002) following a previously established procedure (Williams et al., 2012). A weighed mass of subsample with a target mass of 2.5 g were placed in ceramic crucibles with lids and brought to 550 °C using a Thermofisher Thermolyne muffle furnace for one hour, and then allowed to cool before being re-weighed. Bulk elemental composition was determined by X-ray Fluorescence using a PANalytical Epsilon 3XLE Series XRF, where accuracy and precision was monitored using NIST soil standards 1646a, 2586, and 2587. Analytical error for relevant trace metals is as follows: Ti (± 0.011 wt.%), Cr (± 2.8 mg/kg), Mn (± 24 0.5 mg/kg), Cu (± 14 mg/kg), Zn (± 4 mg/kg), and Pb (± 30 mg/kg). Pellets were made by mixing 11 g of milled soil and 1.2 g of cellulose binder (SPEX 3642) and then pressed to 20 tons of pressure twice for one minute using a Carver 3664 pellet press. Bulk mineralogical composition was determined using a Rigaku Miniflex 6G X-ray Diffractometer. Diffraction scans were conducted at 40 kV and 15 Amps, and a range of 2θ from 3.0 to 90.0°. Rigaku’s PDXL software, with the Whole Pattern Powder Fitting (WPPF) method with access to the International Centre for Diffraction Data (ICDD) database, was used for phase identification and percent abundance determination. Grain-scale characterization to determine morphology and composition was conducted using a Hitachi Bench top Scanning Electron Microscope (SEM) TM3030. The SEM was equipped with Quantax70 energy dispersive x-ray spectrometry (SEM–EDS) operated at 15 kV voltage, and a detector-to-sample working distance typically of 8 mm.

### Acid-extractable metal concentrations (MAE) and Pb isotopic composition

The labile fraction of Pb and other metals was determined by treating the milled soil samples with nitric acid (HNO_3_) (Erel et al., 1997), using 1 g of milled soil mixed with 10 mL of 0.5 M HNO_3_. A preliminary analysis of a subset of these soils comparing the nitric acid extraction to US EPA Method 1340 (i.e. a gastric acid assay) found similar percentages of extractable Pb (Santoro, 2020); the nitric acid extraction was used for all samples to be consistent with previous work that focused on isotopic analysis of labile Pb (Zohar et al., 2014, 2017). Each sample was agitated at 30 rpm for 2 h on an end-over-end rotator. Samples were then centrifuged for 10 min and the supernatant was filtered (0.45 μm) and stored at 2 °C in 15 mL polyethylene bottles. All reagents used were analytical grade and solutions were prepared with distilled-deionized water (DDI-H_2_O) (18.2 MΩ; Milli-Q Direct-Q 3UV-R). Procedural blanks and certified standards were analyzed along with the samples during subsequent analyses. Major and minor element (Mg, Al, Si, P, K, Ti, Mn, and Fe) concentrations were determined by Inductively Coupled Plasma Optical Emission Spectroscopy (ICP-OES, Perkin Elmer Optima 3300) and minor element (Cr, Co, Ni, Cu, Zn, As, Cd, Pb, and U) concentrations were determined by Inductively Coupled plasma Mass Spectrometry (ICP-MS, Perkin-Elmer NexION 300D). Ten randomly selected samples were treated as duplicates; differences between duplicate sub-samples and repeated standards were lower than 5% for all elements.

The Pb isotopic composition of the Pb_AE_ fraction was determined using a multi-collected ICP-MS (MC-ICP-MS, Nu Instruments) (Ehrlich et al., 2001; Platzner et al., 2001) on samples that were treated by column separation of Pb (Ehrlich et al., 2004; Zohar et al., 2014, 2017). Repeated measurements of a Pb isotope standard (NIST SRM 981) were conducted for accuracy and precision control. The 2σ reproducibility on the SRM 981 measurements are 0.17‰ (^208^Pb/^204^Pb), 0.13‰ (^207^Pb/^204^Pb), and 0.07‰ (^206^Pb/^204^Pb).

## Results

### Relationship MT and MAE and neighborhood distribution

**Fig. 2:**
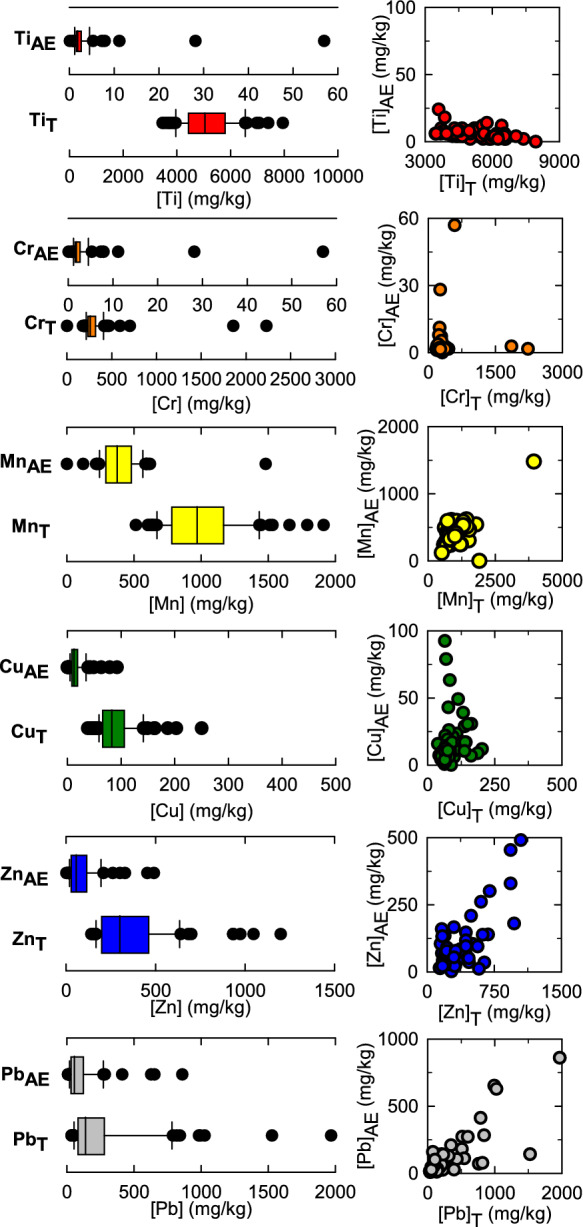
Box-and-whisker plots (left) for [M]_T_ and [M]_AE_ for M = Ti, Cr, Mn, Cu, Zn, and Pb (n = 82), and scatter plots (right) showing the relationship between [M]_T_ and [M]_AE_

The range of [M_T_] and [M_AE_] are shown for trace metals for which both XRF and ICP-MS data was collected (Fig. 2). In general, there was a broad range of [M]_T_ values, particularly for Pb and Zn which ranged over three orders of magnitude. With respect to Pb, 70 soil samples (85%) had [Pb]_T_ above background concentrations (50 mg/kg, for Cuyahoga County) (Christman et al., 2013) and 14 samples (17%) had [Pb]_T_ concentrations above the EPA’s action of 400 mg/kg. Values for [Pb]_T_ in the Summit Lake and West Akron ranged from 52 to 600 mg/kg and 34 to 1970 mg/kg, with 12 of the 14 highest [Pb]_T_ values in West Akron (Fig. 1). With regard to other trace metals, 100% of the samples had above background concentrations for Cr (19 mg/kg), Mn (301 mg/kg), Cu (37 mg/kg), and Zn (91 mg/kg) (Christman et al., 2013), with a median Zn_T_ value (300 mg/kg) three times higher than background concentrations.

Acid-extractable concentration values of the trace metals exhibited similar trends and broad ranges compared to [M]_T_, particularly for Pb and Zn which ranged over two orders of magnitude (Fig. 2). Linear correlations were observed between [M]_T_ and [M]_AE_ for Pb and Zn, with R^2^ values of 0.66 and 0.55, respectively (SI Table 1) and corresponds to a percentage of acid-extractable Pb and Zn of 35% and 33%, respectively. There were no apparent differences in metal lability between the two neighborhoods; for example, Pb_AE_ was 36% of Pb_T_ in Summit Lake (R^2^ of 0.72) and 35% in West Akron (R^2^ of 0.65). The other trace metals (Ti, Cr, Mn, and Cu) did not exhibit a linear correlation between [M]_T_ and [Mn]_AE_ (Fig. 2). With respect to the geographic distribution of [Pb_AE_], higher average [Pb]_AE_ values were observed in West Akron (174 mg/kg) compared to Summit Lake (80 mg/kg), consistent with the observed [Pb_T_] distribution (Fig. 1).

Pearson correlation values (SI Table 1) indicate that Pb and Zn exhibited a strong positive correlation with each other in both the M_T_ and M_AE_ fractions (R^2^ = 0.56 and 0.68, respectively), with no significant differences in the correlations between the two neighborhoods. Further, Pb_AE_ also exhibited moderate positive correlations with K_AE_ and Cd_AE_, and a moderate weak correlation with Si_T_. Additional correlations for other trace metals were also observed (SI Table 1): Ti_AE_ had a strong positive correlation with Cu_T_ and Si_AE_; Cr_AE_ had a strong positive correlation with P_AE_; Mn_AE_ had a strong positive correlation with Ca_T_, Mg_T_, Al_AE_, and Ca_AE_; Co_AE_ had a strong positive correlation with V_AE_; and Ni_AE_ had a strong positive correlation with Cu_T_, Mg_AE_, Si_AE_, and Ca_AE_. The relationship between these correlations and observed solid phase speciation, with an emphasis on Pb, will be explored below.

### Characterization of Pb-bearing solids and soil properties

Soil mineralogy was dominated by quartz, feldspars (albite and anorthite), and phyllosilicates (kaolinite, muscovite, and biotite), with average values of 85% ± 9%, 3% ± 5%, and 11% ± 8%, respectively (SI Figure 1A); no Pb-bearing phases were detected by XRD. The soil samples were observed to have a sandy texture following air-drying, consistent with the high quartz context determined by XRD. Further, loss-on-ignition values were generally low (less than 1.5%), with an average value of 0.41% ± 0.26%. (SI Figure 1A).

**Fig. 3:**
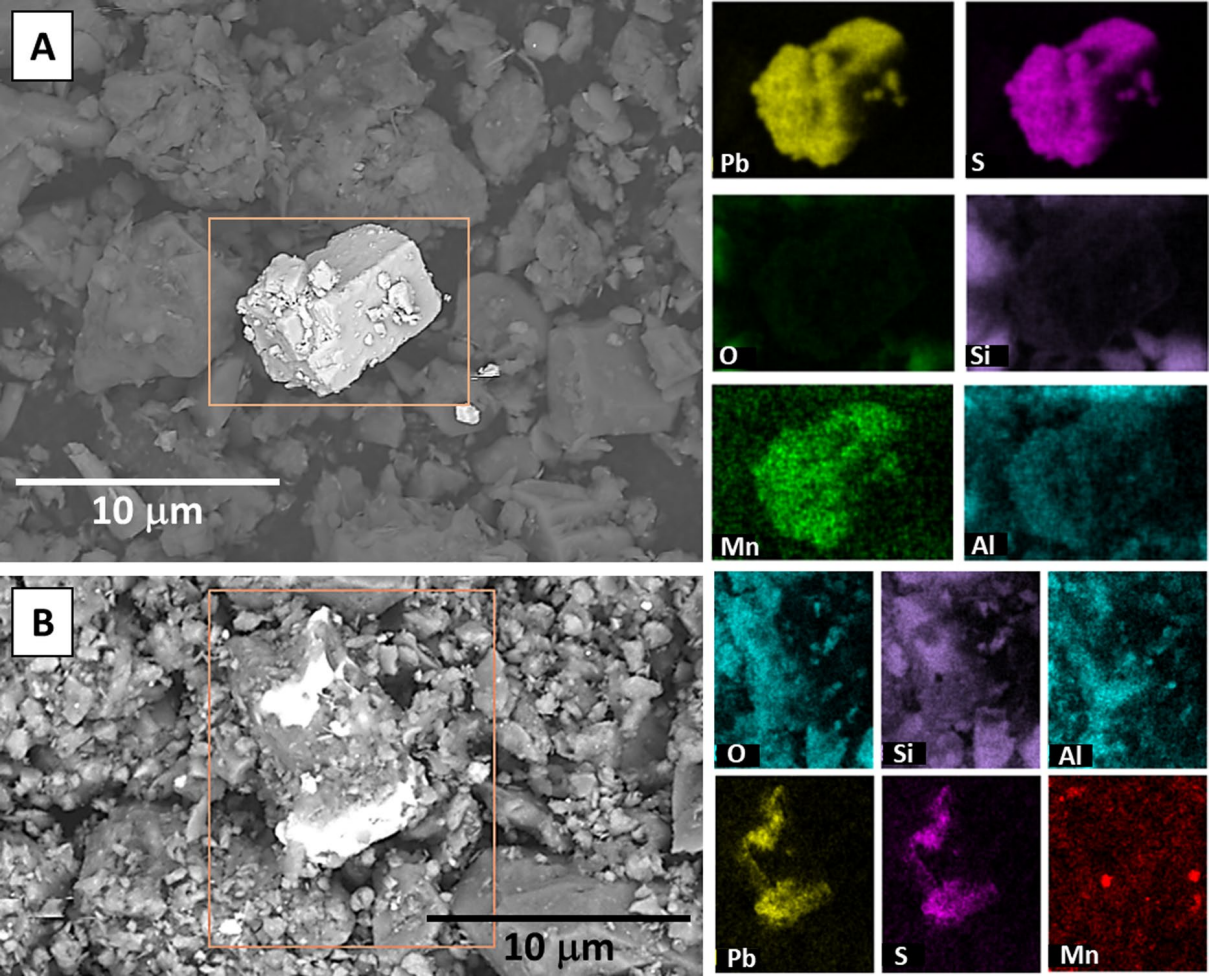
Representative SEM images and EDS element maps for two samples showing Pb–S-rich phases consistent with the composition and morphology of galena. Values for [Pb]_T_ and [Pb]_AE_ were 263 mg/kg and 26 mg/kg, respectively (**A**) and 762 mg/kg and 114 mg/kg, respectively (**B**)

**Fig. 4:**
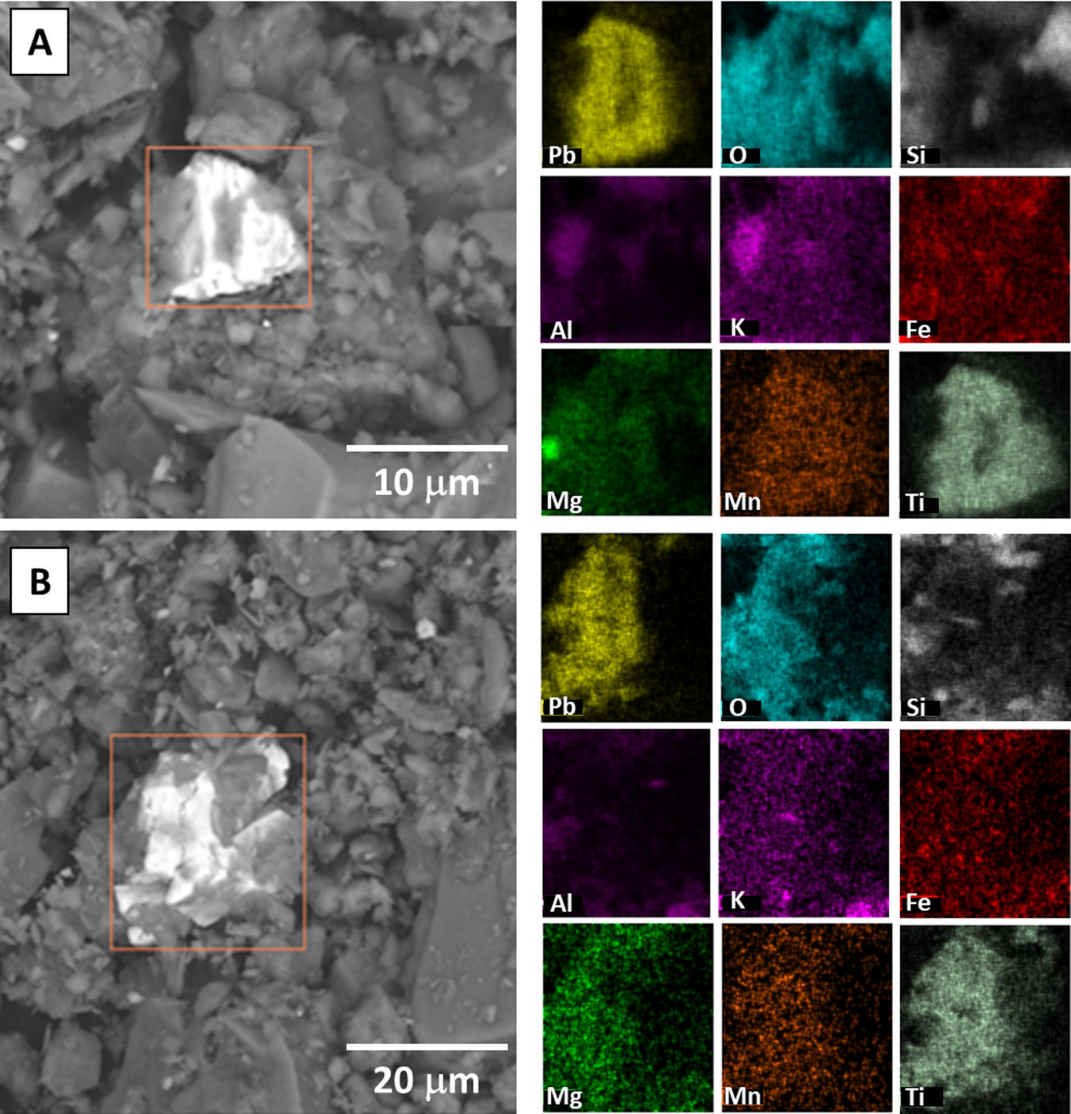
Representative SEM images and EDS maps of Pb-Ti–O-rich phases consistent with paint particles. Values for [Pb]_T_ and [Pb]_AE_ were 544 mg/kg and 123 mg/kg, respectively (**A**) and 517 mg/kg and 102 mg/kg, respectively (**B**)

**Fig. 5:**
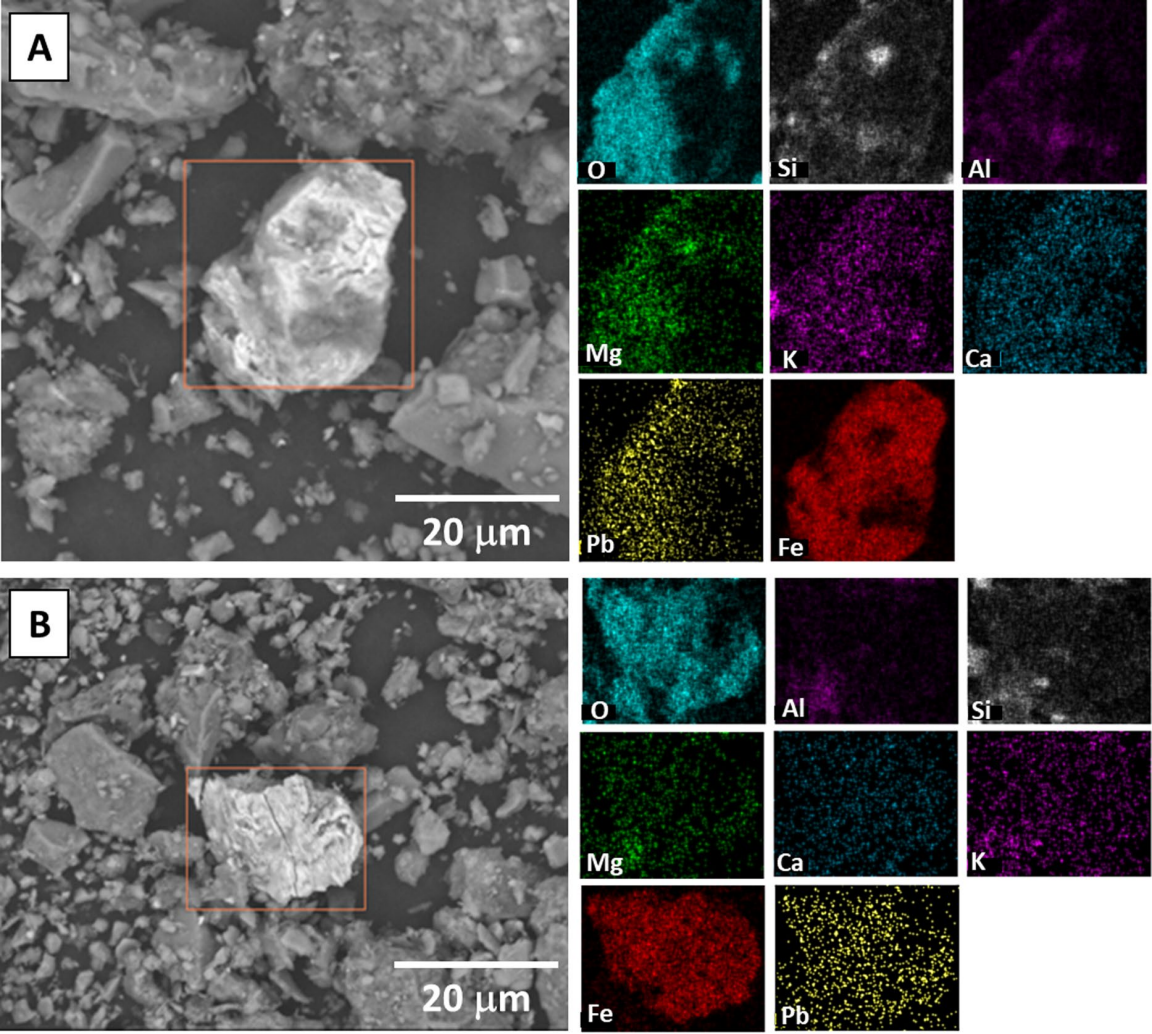
Representative SEM images and EDS maps of Pb-Fe–O-rich phases consistent with Pb associated with Fe-oxides. Values for [Pb]_T_ and [Pb]_AE_ were 435 mg/kg and 260 mg/kg, respectively (**A**) and 139 mg/kg and 79 mg/kg, respectively (**B**)

Grain-scale characterization of 20 randomly selected samples (ten from each neighborhood) by SEM–EDS analyses revealed the presence of three types of Pb-bearing phases, which were readily apparent based on their high contrast in the SEM images, despite their low modal abundance compared to the (alumino)silicate minerals that dominate the soil mineralogy. The Pb-bearing phases are described in order of highest to lowest Pb concentration of the analyzed grains based on EDS spot analyses, however, no correlation was observed between the presence of high-concentration Pb-bearing phases and the [Pb]_T_ or [Pb]_AE_ values of the analyzed soil sample. (1) Pb–S-rich cubic phases, consistent with the composition and morphology of galena (PbS), and 5–10 µm in size (Fig. 3 and SI Figure 2). Some of the Pb–S-rich grains were spatially correlated with other trace metals including Mn (Fig. 3A) and Zn (SI Figure 2B), and coated in Si-Al-O rich phases consistent with the composition and texture of clays (Fig. 3B) or Fe–O-rich phases consistent with Fe-oxides (SI Figure 2A). The Pb–S-rich grains were observed in five of the 20 analyzed samples, and only in the Summit Lake neighborhood samples. (2) Pb–O-Ti–rich phases with angular morphology that were 10–20 µm in size, spatially correlated with low concentrations of Mg and Mn, and coated and/or in aggregates of fine-grained phases consistent with the composition (Si-Al–Mg-O) and texture of clays (Fig. 4). These Pb-bearing phases were observed in four of the 20 analyzed samples, and present in samples from both the Summit Lake and West Akron samples. (3) Pb-Fe–O-rich phases with angular morphology that were 20 µm in size, and consistent with Pb-bearing Fe-oxides and were not typically spatially correlated with other trace metals (Fig. 5). These Pb-bearing phases were observed in five of the 20 analyzed samples, and present in samples from both the Summit Lake and West Akron samples. After completing the acid extraction experiment, four randomly selected samples (two from each neighborhood) were also analyzed by SEM–EDS, and no discrete Pb-bearing phases were observed in those samples (SI Figure 3). In both pre- and post-acid extraction samples, the presence of other anthropogenic phases was also observed (SI Figure 3), primarily spherical particles 20 µm in diameter or aggregates of spherical particles 100 µm in size and consistent with metallic slag.

### Isotopic composition of acid-extractable Pb

**Fig. 6:**
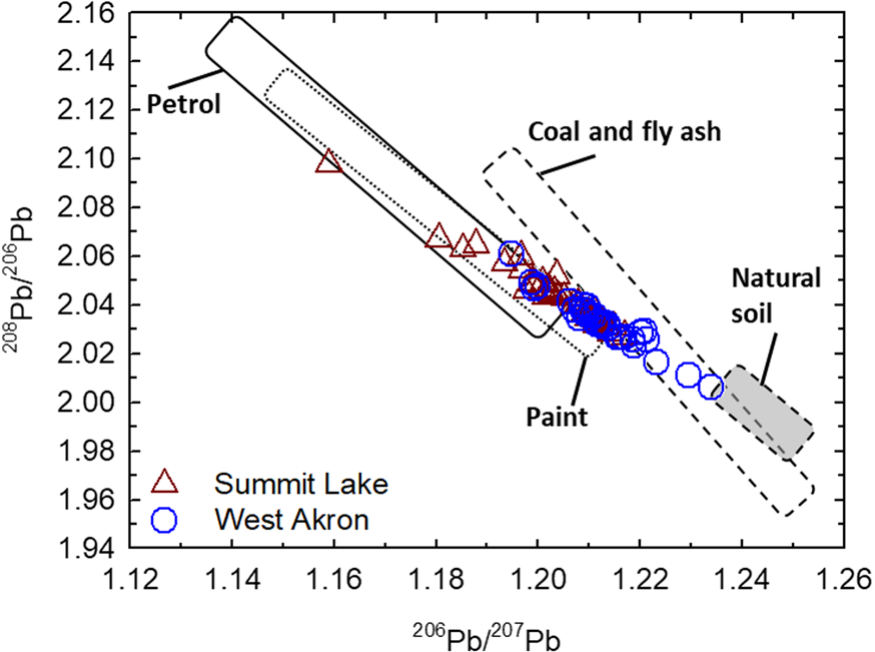
Isotopic composition of acid-extractable Pb (Pb_AE_) for all Akron soil samples separated by neighborhood (red triangles for Summit Lake; and blue circles for West Akron). Also included are average data and ranges for US soil (natural Pb, gray area), paint (short dash-lined area), petrol (solid-lined region), and coal and fly ash (long dashed-line area) (Wang et al., 2019). Error bars (representing instrumental error based on reference standards collected in between samples) are smaller than the data symbols

Isotope ratio values for all acid-extractable Pb samples were 1.159 to 1.245 for ^206^Pb/^207^Pb, and 1.999 to 2.098 for ^208^Pb/^206^Pb (Fig. 6). Although there was some overlap in the Pb isotope ratio values of the Summit Lake and West Akron neighborhood soil samples, there was a statistically significant difference between the two datasets based on a t-test analysis (*p* = 0.010 for ^206^Pb/^207^Pb and *p* = 0.009 for ^208^Pb/^206^Pb). Isotope ratio values for the Summit Lake soil samples were 1.159 to 1.217 for ^206^Pb/^207^Pb, and 2.027 to 2.098 for ^208^Pb/^206^Pb; value ranges for West Akron were 1.195 to 1.234 for ^206^Pb/^207^Pb, and 2.006 to 2.061 for ^208^Pb/^206^Pb. The isotopic ratios of the acid extracted Pb were compared to Pb sources to determine major contributors to labile Pb in the Akron soil samples. Potential sources include uncontaminated soil, leaded gasoline (i.e., petrol), lead paint, and coal and fly ash ((Wang et al., 2019), and references therein). The Pb isotope values from the Akron soil samples fall primarily within the paint and petrol ranges, primarily in the higher ^206^Pb/^207^Pb and lower ^208^Pb/^206^Pb range of source values (Fig. 6). Half of the Summit Lake samples fall outside of the petrol range and are clustered near the end of the paint range, and a third of the West Akron samples fall outside of both the petrol and paint ranges and are within the coal and fly ash range.

## Discussion

### Comparison of total versus labile Pb in Akron and other cities

The range of [Pb]_T_ values in the current study (34 mg/kg to 1970 mg/kg) (Fig. 2) are similar to previous studies of urban areas with comparable sampling densities, including Los Angeles, California, USA (9 mg/kg to 8150 mg/kg Pb_T_, n = 550) (Wu et al., 2010); Lithgow, New South Wales, Australia (b.d.l. to 3490 mg/kg Pb_T_, n = 134) (Rouillon et al., 2013); and New Castle, New South Wales, Australia (14 mg/kg to 11,600 mg/kg, n = 170) (Harvey et al., 2017); Toledo, Ohio, USA (b.d.l. to 904 mg/kg Pb_T_, n = 81) (Stewart et al., 2014); Philadelphia, Pennsylvania, USA (58 mg/kg to 2821 mg/kg, n = 38) (Bradham et al., 2017); Greensboro, North Carolina, USA (b.d.l. to 1200 mg/kg, n = 2310) (Obeng-Gyasi et al., 2021), and Indianapolis, Indiana, USA ( b.d.l. to 6619 mg/kg Pb_T_, n = 1400) (Filippelli et al., 2018). The results of the current work indicate that, like these other urban locations, Akron has elevated soil Pb levels likely due to presence of older housing stock and history of leaded gasoline use. Other industrial activities that produce Pb emissions had a more limited presence in Akron (e.g. smelting, glass working, production and joining of pipes, match production, etc.), although these could have resulted in additional localized Pb contamination.

Although previous efforts aiming to determine the fraction of Pb_T_ that is potentially bioaccessible in urban residential soils have used different extraction protocols, it is clear that in contrast to the range and average values of Pb_T_, the amount of potentially bioaccessible Pb is quite variable. For example, in the current work, approximately 35% of Pb_T_ was acid-extractable (Fig. 3), in contrast to previous studies which reported a labile fraction of approximately 60–70% (Hamel et al., 1999; Juhasz et al., 2009; Paltseva et al., 2018; Stewart et al., 2014; Wu et al., 2010) and up to 80–90% (Bradham et al., 2017; Lamb et al., 2009), although average values across a wide range of urban and residential soils has been reported to be approximately 50% (Yan et al., 2017). The percent of labile Pb in materials from non-residential soils vary more widely from 10 to 90%, and includes shooting range soils, incinerator waste, former landfill, and smelter waste (Smith et al., 2011), and more broadly is dependent on soil type and soil properties (Yan et al., 2017). The lower fraction of labile Pb in the current work might reflect slight differences in the analytical approach and/or in sources and local changes in Pb speciation over time, including variability in the proportional input of Pb from historical gasoline versus paint sources. For example, Los Angeles has a much higher road and traffic density compared to Akron coupled with lower rates of Pb paint use which might account for the higher amounts of potentially bioaccessible Pb (Wu et al., 2010). Further, following deposition of Pb to soils, the amount of potentially bioaccessible Pb has been shown to decrease over time due to aging reactions and changes in speciation, with an increase in the fraction of Pb associated with Fe- and Mn-oxides (Saminathan et al., 2010). If Pb inputs to Akron soils are dominated by older sources (i.e., paint from houses in the 1950s) compared to newer inputs (i.e., gasoline emissions through the 1970s) then it is possible that changes in Pb speciation in Akron soils over time has resulted in an increase in the proportion of recalcitrant Pb compared to other cities with different Pb emission sources and time profiles. The lower fraction of labile Pb in the current work could also be due to and overestimation of total Pb by XRF and/or an underestimation of potentially bioaccessible Pb during the acid extraction and further work is warranted on a wider range of samples from the study site. Ultimately, these results highlight the need to determine Pb speciation in soils to better ascertain the relationship between total and labile Pb.

### Factors controlling the presence of primary and secondary Pb-bearing phases in Akron soils

The speciation of Pb in the Akron soil samples is complex and is likely the result of a combination of multiple source inputs combined with post-depositional transformations. Given that no discrete Pb-bearing phases were observed in the post-acid extracted samples (SI Figure 3), the three type of Pb-bearing phase observed by SEM–EDS likely dominate the fraction of labile Pb. Soils from both Akron neighborhoods contained Pb-bearing particles that were compositionally consistent with particles of Pb-pigments in paint and present in aggregates with other common phases in historical Pb-based paint (Fig. 4). The composition of white Pb-pigments, the primary Pb paint used in residential neighborhoods, included Pb-oxides, Pb-carbonate, and Pb-sulfate (Beauchemin et al., 2011; Hunt, 2016). Non-Pb phases include other pigments (modifying paint color such as Ti- and Zn-oxides), or as fillers (modifying the physical characteristics of the paint, including clays, quartz, and carbonates Dietrich et al., 2022; Hunt, 2016). The presence of these phases is consistent with a high density of older housing stock in Akron with historical Pb-paint use (Schuch et al., 2017).

The association of Pb with Fe-oxides (Fig. 4) is commonly observed in soils, where the addition of Pb^2+^ added to soils from a variety of sources, dominated by paint and gasoline emissions, typically results in the retention of Pb in soils through sequestration by metal oxides, clays, and organic matter (Mielke et al., 2019; Wade et al., 2021). Following deposition of Pb-paint into soils through chipping or peeling from older structures, common pigments (e.g. Pb-oxides and Pb-carbonates) undergo weathering and are typically transformed into soluble Pb-hydroxides which subsequently dissolve and release Pb^2+^ (Davis et al., 1993). An additional source of Pb^2+^ was from deposition of leaded gasoline emissions, where combustion of organo-Pb compounds resulted in the formation of Pb^2+^ (Seyferth, 2003). Sequestration of Pb^2+^ by Fe-oxides has been observed in in soils where gasoline emissions were dominant with no input from paint (Kaste et al., 2006), in soils where paint is the primary source of Pb (Clark et al., 2006), and in soils with multiple Pb source inputs (Haque et al., 2021). In the current work, the Fe-oxides Pb was associated with were observed to be correlated with Mn as well (Fig. 4). Although Pb was not observed to be correlated with discrete Mn-oxides, these phases can be important sorbents of Pb^2+^ in soils (O’Reilly & Hochella, 2003).

The presence of galena-like phases in the Akron soils (Fig. 3), although in low abundance, is surprising given the lack of Pb-mining activities, smelters, or other industries that specifically used galena in the area. Given the industrial history of Akron, one possible explanation for the presence of galena is the result of the reaction between labile Pb-bearing phases and sulfur emissions from nearby rubber and tire manufacturing facilities. It has been documented since the 1940s that the presence of S in the atmosphere (e.g. near mining towns and sewage plants) can react with Pb paint pigments (e.g. Pb-oxides and carbonates) forming Pb sulfide with subsequent darkening of paint (Sperry, 1948). Rubber production, specifically the creation of latex, is also known to produce hydrogen sulfide which was vented directly to the atmosphere to decrease worker exposure (McCormick, 1952). These emissions were linked to numerous health and safety issues in the rubber industry in the 1960s, including acute eye irritation and chronic vision impairment among factory workers, and air pollution in adjacent neighborhoods (Brown, 1969). It is possible that Pb-bearing particles from paint pigments in soils could react with S emissions from the Akron rubber industries to produce galena. Further, the rubber and tire factories were located in the southeastern portion of Akron, and the Summit Lake neighborhood is adjacent to these locations whereas West Akron is 5–10 km away. This may explain why particles of PbS were only observed in Summit Lake which would have been closer to the S emission sources. Further work is warranted to quantify the abundance of the galena-like phases using synchrotron-based bulk X-ray Absorption Spectroscopy, which coupled with Pb isotopic analyses can aid in quantifying the primary sources of contamination (e.g. (Noerpel et al., 2020)).

### Variability in legacy Pb sources at the neighborhood scale

Lead isotope ratio values (Fig. 6) indicate that the majority of samples from both neighborhoods fall within the leaded gasoline and paint isotopic ratio values, which is consistent with the similarity in the age of housing stock (pre-1950) (Schuch et al., 2017) and road densities of the two neighborhoods that would result in similar Pb sources dominating Pb input to urban soils (Clark et al., 2006). However, approximately half of the West Akron samples fall outside of the gasoline range and are clustered in the lower range of the paint range. This difference potentially reflects where the samples were collected; all samples from Summit Lake were collected at road verges, whereas the West Akron samples were collected by students from homes, nearby parks, or schoolyards throughout Akron. Previous work has shown soils closer to homes with a history of leaded paint use had Pb isotopic ratio values dominated by Pb paint whereas soils closer to streets had values dominated by legacy leaded gasoline as well as mixed sources from atmospheric deposition (Resongles et al., 2021; Wang et al., 2022). Some studies have shown that in locations where Pb was derived from both paint and gasoline, the isotopic signature of gasoline derived Pb was be dominant (MacKinnon et al., 2011; Takaoka et al., 2006), whereas others have shown that high Pb in soils is mainly derived from the legacy of lead-based paint (Wade et al., 2021; Wang et al., 2022).

Although historical leaded paint and gasoline emissions were expected to be the dominant sources of Pb to both neighborhoods, half of the West Akron samples fall outside of the Pb isotope ratio range for both gasoline and paint (Fig. 6). These values are within the Pb isotopic range of fly ash, generated either from coal combustion or burning of municipal waste (Li et al., 2017; Wang et al., 2021). The West Akron neighborhood is located closer to historical point source emission sources from smoke stacks, including the Ohio Edison Steam Power Plant built in 1954 and the Recycle Energy System Plant that was built to replace it in 1976, which was known to emit lead and other toxic elements and was one of biggest point source polluters in Ohio (Bergin et al., 2008). Further, air pollution from these point sources was found to primarily impact neighborhoods closer to the smokestacks (Mostardi et al., 1981), which could explain why soils in West Akron contain Pb with an isotopic signature from fly ash. Further complications linking sources of Pb based on isotopic composition are changes in the isotopic composition of Pb in leaded gas over the twentieth century (Dunlap et al., 2000; Hurst et al., 1996) and remobilization and mixing Pb in soils, sediments, and aerosols resulting in isotopically mixed anthropogenic Pb (Bollhöfer & Rosman, 2001, 2002; Dietrich et al., 2022; Jaeger et al., 1998). Ultimately, despite some complexity in source apportionment, the Pb isotope signature in Akron soils is dominated by historical paint and gasoline use, consistent with the industrial history of Akron.

## Conclusions and implications

We have shown that Pb speciation and distribution at the neighborhood-scale in Akron, Ohio is variable and reflects a complex local relationship between the input of primary sources and secondary reactions that take place following deposition. Although a correlation between total and labile Pb is expected, localized variability in soil type and secondary reactions makes it difficult to predict the fraction of potentially bioaccessible Pb. Akron soil samples are contaminated with Pb and although the labile fraction is a lower percentage compared to studies in other cities, the high total concentrations result in significant source of labile, and potentially bioaccessible Pb. Our work is consistent with previous studies that have shown that urban core properties represent the greatest Pb exposure risk, particularly soils near older homes (Filippelli et al., 2018). Further, by linking neighborhood-dependent Pb speciation and distribution to previous work that has linked older housing stock to elevated BLLs among children in Akron (Schuch et al., 2017), and this work highlights the importance of community science collaborations to expand the reach of soil sampling and establish areas most at risk based on neighborhood-dependent Pb speciation and distribution for targeted remediation. The current system in place for discovering and treating elevated BLLs is reactive rather than proactive. The use of a proactive community science collaborations allows for the identification of high concentrations, so they can be addressed before allowing families to establish residency and/or advocate for remedial actions (Dietrich et al., 2023a, 2023b).

The pervasiveness of labile and potentially bioaccessible Pb across Akron indicates that remediation targeting areas with highest Pb concentrations in residential areas is necessary, which could include covering contaminated urban soils and/or treating them to convert Pb to more recalcitrant phases, both of which are expensive over large areas but will cost much less than treating impacted children (Karna et al., 2021; Mielke et al., 2006; Sowers et al., 2021). Further, without treatment, these soils can potentially be a persistent source of airborne Pb resulting in an additional exposure pathway (Harlavan et al., 2020; Resongles et al., 2021; Wang et al., 2022). Further work is warranted to determine the distribution of labile Pb over the wider metropolitan Akron area and determine the speciation and distribution of Pb in aerosols suspended above the area.

### Supplementary Information

Below is the link to the electronic supplementary material.

Supplementary file1 (XLSX 38 KB)

Supplementary file2 (DOCX 820 KB)

## Data Availability

The authors declare that the data supporting the findings of this study are available within the paper and its supplementary information files.

## References

[CR1] Alloway BJ (2013). Sources of heavy metals and metalloids in soils Heavy metals in soils.

[CR2] Beauchemin S, MacLean LCW, Rasmussen PE (2011). Lead speciation in indoor dust: A case study to assess old paint contribution in a Canadian urban house. Environmental Geochemistry and Health.

[CR3] Bergin MS, Russell AG, Odman MT, Cohan DS, Chameides WL (2008). Single-source impact analysis using three-dimensional air quality models. Journal of the Air & Waste Management Association.

[CR4] Bi X, Liang S, Li X (2013). A novel in situ method for sampling urban soil dust: Particle size distribution, trace metal concentrations, and stable lead isotopes. Environmental Pollution.

[CR5] Biggins PD, Harrison RM (1980). Chemical speciation of lead compounds in street dusts. Environmental Science and Technology.

[CR6] Bollhöfer A, Rosman K (2001). Isotopic source signatures for atmospheric lead: The northern hemisphere. Geochimica Et Cosmochimica Acta.

[CR7] Bollhöfer A, Rosman K (2002). The temporal stability in lead isotopic signatures at selected sites in the southern and northern hemispheres. Geochimica Et Cosmochimica Acta.

[CR8] Bradham KD, Laird BD, Rasmussen PE, Schoof RA, Serda SM, Siciliano SD, Hughes MF (2014). Assessing the bioavailability and risk from metal-contaminated soils and dusts. Human and Ecological Risk Assessment: An International Journal.

[CR9] Bradham KD, Nelson CM, Kelly J, Pomales A, Scruton K, Dignam T, Misenheimer JC, Li K, Obenour DR, Thomas DJ (2017). Relationship between total and bioaccessible lead on children’s blood lead levels in urban residential philadelphia soils. Environmental Science and Technology.

[CR10] Brown K (1969). Some toxicological problems in a rubber industry. Medical Journal of Australia.

[CR11] CDC (2012) Low level lead exposure harms children: A renewed call for primary prevention. Report of advisory committee on childhood lead poisoning prevention. *Atlanta: Centers for Disease Control*

[CR12] Chow TJ, Johnstone MS (1965). Lead isotopes in gasoline and aerosols of Los Angeles Basin, California. Science.

[CR13] Christman, T., Martin, J., Myers, F., Rasik, C., Sainey, E., & Steigerwald-Dick, V. (2013). Evaluation of background metal soil concentrations in Cuyahoga county – Cleveland area, OH-EPA summary report. Ohio EPA

[CR14] Clark HF, Brabander DJ, Erdil RM (2006). Sources, sinks, and exposure pathways of lead in urban garden soil. Journal of Environmental Quality.

[CR15] Davis A, Drexler JW, Ruby MV, Nicholson A (1993). Micromineralogy of mine wastes in relation to lead bioavailability, Butte Montana. Environmental Science & Technology.

[CR16] Dean WE (1974). Determination of carbonate and organic matter in calcareous sediments and sedimentary rocks by loss on ignition; comparison with other methods. Journal of Sedimentary Research.

[CR17] Dietrich M, Rader ST, Filippelli GM (2023). Using community science for detailed pollution research: A case-study approach in Indianapolis, IN, USA. Environmental Science and Pollution Research.

[CR18] Dietrich M, Shukle JT, Krekeler MPS, Wood LR, Filippelli GM (2022). Using community science to better understand lead exposure risks. GeoHealth.

[CR19] Dietrich M, Wood LR, Shukle JT, Herrmann A, Filippelli GM (2023). Contributory science reveals insights into metal pollution trends across different households and environmental media. Environmental Research Letters.

[CR20] Dunlap CE, Bouse R, Flegal AR (2000). Past leaded gasoline emissions as a nonpoint source tracer in riparian systems: A study of river inputs to San Francisco bay. Environmental Science and Technology.

[CR21] Egan KB, Cornwell CR, Courtney JG, Ettinger AS (2021). Blood lead levels in US children ages 1–11 years, 1976–2016. Environmental Health Perspectives.

[CR22] Ehrlich S, Ben-Dor L, Halicz L (2004). Precise isotope ratio measurement by multicollector-ICP-MS without matrix separation. Canadian Journal of Analytical Sciences and Spectroscopy.

[CR23] Ehrlich S, Karpas Z, Ben-Dor L, Halicz L (2001). High precision lead isotope ratio measurements by multicollector-ICP-MS in variable matrices. Journal of Analytical Atomic Spectrometry.

[CR24] Erel Y, Veron A, Halicz L (1997). Tracing the transport of anthropogenic lead in the atmosphere and in soils using isotopic ratios. Geochimica Et Cosmochimica Acta.

[CR25] Filippelli GM, Adamic J, Nichols D, Shukle J, Frix E (2018). Mapping the urban lead exposome: A detailed analysis of soil metal concentrations at the household scale using citizen science. International Journal of Environmental Research and Public Health.

[CR26] Filippelli GM, Risch M, Laidlaw MA, Nichols DE, Crewe J (2015). Geochemical legacies and the future health of cities: A tale of two neurotoxins in urban soils. Elementa.

[CR27] Flora G, Gupta D, Tiwari A (2012). Toxicity of lead: A review with recent updates. Interdisciplinary Toxicology.

[CR28] Geller MD, Ntziachristos L, Mamakos A, Samaras Z, Schmitz DA, Froines JR, Sioutas C (2006). Physicochemical and redox characteristics of particulate matter (PM) emitted from gasoline and diesel passenger cars. Atmospheric Environment.

[CR29] Gilbert SG, Weiss B (2006). A rationale for lowering the blood lead action level from 10 to 2 μg/dL. Neurotoxicology.

[CR30] Hamel SC, Ellickson KM, Lioy PJ (1999). The estimation of the bioaccessibility of heavy metals in soils using artificial biofluids by two novel methods: Mass-balance and soil recapture. Science of the Total Environment.

[CR31] Haque E, Thorne PS, Nghiem AA, Yip CS, Bostick BC (2021). Lead (Pb) concentrations and speciation in residential soils from an urban community impacted by multiple legacy sources. Journal of Hazardous Materials.

[CR32] Harlavan Y, Shirav M, Ilani S, Halicz L, Yoffe O (2020). The fate of Anthropogenic Pb in soils; years after Pb terminated as a fuel additive Northern Israel. Environmental Pollution.

[CR33] Harvey PJ, Rouillon M, Dong C, Ettler V, Handley HK, Taylor MP, Tyson E, Tennant P, Telfer V, Trinh R (2017). Geochemical sources, forms and phases of soil contamination in an industrial city. Science of the Total Environment.

[CR34] Hauptman M, Bruccoleri R, Woolf AD (2017). An update on childhood lead poisoning. Clinical Pediatric Emergency Medicine.

[CR35] Hunt A (2016). Relative bioaccessibility of Pb-based paint in soil. Environmental Geochemistry and Health.

[CR36] Hurst RW, Davis TE, Chinn BD (1996). Peer reviewed: The lead fingerprints of gasoline contamination. Environmental Science and Technology.

[CR37] Hwang H-M, Fiala MJ, Park D, Wade TL (2016). Review of pollutants in urban road dust and stormwater runoff: Part 1. Heavy metals released from vehicles. International Journal of Urban Sciences.

[CR38] Jaeger RJ, Weiss AL, Manton WI (1998). Isotopic ratio analysis in residential lead-based paint and associated surficial dust. Journal of Toxicology: Clinical Toxicology.

[CR39] Juhasz AL, Weber J, Smith E (2011). Impact of soil particle size and bioaccessibility on children and adult lead exposure in peri-urban contaminated soils. Journal of Hazardous Materials.

[CR40] Juhasz AL, Weber J, Smith E, Naidu R, Marschner B, Rees M, Rofe A, Kuchel T, Sansom L (2009). Evaluation of SBRC-gastric and SBRC-intestinal methods for the prediction of in vivo relative lead bioavailability in contaminated soils. Environmental Science and Technology.

[CR41] Karna RR, Noerpel MR, Nelson C, Elek B, Herbin-Davis K, Diamond G, Bradham K, Thomas DJ, Scheckel KG (2021). Bioavailable soil Pb minimized by in situ transformation to plumbojarosite. Proceedings of the National Academy of Sciences.

[CR42] Kaste JM, Bostick BC, Friedland AJ, Schroth AW, Siccama TG (2006). Fate and speciation of gasoline-derived lead in organic horizons of the northeastern USA. Soil Science Society of America Journal.

[CR43] Konen ME, Jacobs PM, Burras CL, Talaga BJ, Mason JA (2002). Equations for predicting soil organic carbon using loss-on-ignition for north central US soils. Soil Science Society of America Journal.

[CR44] Laidlaw MA, Filippelli GM, Sadler RC, Gonzales CR, Ball AS, Mielke HW (2016). Children’s blood lead seasonality in flint, Michigan (USA), and soil-sourced lead hazard risks. International Journal of Environmental Research and Public Health.

[CR45] Laidlaw MAS, Mohmmad SM, Gulson BL, Taylor MP, Kristensen LJ, Birch G (2017). Estimates of potential childhood lead exposure from contaminated soil using the US EPA IEUBK Model in Sydney Australia. Environmental Research.

[CR46] Laidlaw MAS, Zahran S, Mielke HW, Taylor MP, Filippelli GM (2012). Re-suspension of lead contaminated urban soil as a dominant source of atmospheric lead in Birmingham, Chicago, Detroit and Pittsburgh USA. Atmospheric Environment.

[CR47] Lamb DT, Ming H, Megharaj M, Naidu R (2009). Heavy metal (Cu, Zn, Cd and Pb) partitioning and bioaccessibility in uncontaminated and long-term contaminated soils. Journal of Hazardous Materials.

[CR48] Lanphear BP, Hornung R, Ho M, Howard CR, Eberly S, Knauf K (2002). Environmental lead exposure during early childhood. The Journal of Pediatrics.

[CR49] Lanphear BP, Matte TD, Rogers J, Clickner RP, Dietz B, Bornschein RL, Succop P, Mahaffey KR, Dixon S, Galke W (1998). The contribution of lead-contaminated house dust and residential soil to children’s blood lead levels: A pooled analysis of 12 epidemiologic studies. Environmental Research.

[CR50] Li Y, Zhang H, Shao L-M, He P-J (2017). Tracing source and migration of Pb during waste incineration using stable Pb isotopes. Journal of Hazardous Materials.

[CR51] MacKinnon G, MacKenzie AB, Cook GT, Pulford ID, Duncan HJ, Scott EM (2011). Spatial and temporal variations in Pb concentrations and isotopic composition in road dust, farmland soil and vegetation in proximity to roads since cessation of use of leaded petrol in the UK. Science of the Total Environment.

[CR52] MacLean LCW, Beauchemin S, Rasmussen PE, Zereini F, Wiseman CLS (2011). Application of synchrotron X-ray techniques for the determination of metal speciation in (house) dust particles. Urban airborne particulate matter: origin, chemistry, fate and health impacts.

[CR53] MacLean LCW, Beauchemin S, Rasmussen PE (2011). Lead speciation in house dust from Canadian urban homes using EXAFS, Micro-XRF, and Micro-XRD. Environmental Science and Technology.

[CR54] Markus J, McBratney AB (2001). A review of the contamination of soil with lead: II. Spatial distribution and risk assessment of soil lead. Environment International.

[CR55] McCormick WE (1952). Industrial health problems in the rubber industry. American Industrial Hygiene Association Quarterly.

[CR56] Mielke HW (1999). Lead in the inner cities: Policies to reduce children’s exposure to lead may be overlooking a major source of lead in the environment. American Scientist.

[CR57] Mielke HW, Anderson JC, Berry KJ, Mielke PW, Chaney RL, Leech M (1983). Lead concentrations in inner-city soils as a factor in the child lead problem. American Journal of Public Health.

[CR58] Mielke HW, Gonzales CR, Powell ET, Laidlaw MA, Berry KJ, Mielke PW, Egendorf SP (2019). The concurrent decline of soil lead and children’s blood lead in New Orleans. Proceedings of the National Academy of Sciences.

[CR59] Mielke HW, Gonzales CR, Powell ET, Mielke PW (2017). Spatiotemporal exposome dynamics of soil lead and children’s blood lead pre- and ten years post-Hurricane Katrina: Lead and other metals on public and private properties in the city of New Orleans, Louisiana, U.S.A. Environmental Research.

[CR60] Mielke HW, Powell ET, Gonzales CR, Mielke PW, Ottesen RT, Langedal M (2006). New orleans soil lead (Pb) cleanup using Mississippi river alluvium: need, feasibility, and cost. Environmental Science and Technology.

[CR61] Miguel ED, Llamas JF, Chacón E, Berg T, Larssen S, Røyset O, Vadset M (1997). Origin and patterns of distribution of trace elements in street dust: Unleaded petrol and urban lead. Atmospheric Environment.

[CR62] Mostardi RA, Ely DL, Woebkenberg NR, Richardson B, Jarrett MT (1981). The University of Akron study on air pollution and human health effects I. Methodology, baseline data, and aerometrics. Archives of Environmental Health: An International Journal.

[CR63] Newell RG, Rogers K (2003). The US experience with the phasedown of lead in gasoline. Resources for the Future.

[CR64] Noerpel M, Pribil M, Rutherford D, Law P, Bradham K, Nelson C, Weber R, Gunn G, Scheckel K (2020). Lead speciation, bioaccessibility and source attribution in Missouri’s big river watershed. Applied Geochemistry.

[CR65] Nriagu JO (1999). Clean hands: clair patterson’s crusade against environmental lead contamination.

[CR66] NTP (2012). Monograph on health effects of low-level lead. *National Institutes of Health, National Institute of Environmental Health Sciences, National Toxicology Program*.

[CR67] O’Reilly SE, Hochella MF (2003). Lead sorption efficiencies of natural and synthetic Mn and Fe-oxides. Geochimica Et Cosmochimica Acta.

[CR68] Obeng-Gyasi E, Roostaei J, Gibson JM (2021). Lead distribution in urban soil in a medium-sized city: Household-scale analysis. Environmental Science and Technology.

[CR69] Paltseva A, Cheng Z, Deeb M, Groffman PM, Maddaloni M (2018). Variability of bioaccessible lead in urban garden soils. Soil Science.

[CR70] Pierson WR, Brachaczek WW (1982). Particulate matter associated with vehicles on the road. II. Aerosol Science and Technology.

[CR71] Pingitore NE, Clague JW, Amaya MA, Maciejewska B, Reynoso JJ (2009). Urban airborne lead: X-ray absorption spectroscopy establishes soil as dominant source. PLoS ONE.

[CR72] Platzner I, Ehrlich S, Halicz L (2001). Isotope-ratio measurements of lead in NIST standard reference materials by multiple-collector inductively coupled plasma mass spectrometry. Fresenius’ Journal of Analytical Chemistry.

[CR73] Resongles E, Dietze V, Green DC, Harrison RM, Ochoa-Gonzalez R, Tremper AH, Weiss DJ (2021). Strong evidence for the continued contribution of lead deposited during the 20th century to the atmospheric environment in London of today. Proceedings of the National Academy of Sciences.

[CR74] Rouillon M, Gore DB, Taylor MP (2013). The nature and distribution of Cu, Zn, Hg, and Pb in urban soils of a regional city: Lithgow Australia. Applied Geochemistry.

[CR75] Ruby MV, Schoof R, Brattin W, Goldade M, Post G, Harnois M, Mosby DE, Casteel SW, Berti W, Carpenter M, Edwards D, Cragin D, Chappell W (1999). Advances in evaluating the oral bioavailability of inorganics in soil for use in human health risk assessment. Environmental Science and Technology.

[CR76] Saminathan SK, Sarkar D, Andra SS, Datta R (2010). Lead fractionation and bioaccessibility in contaminated soils with variable chemical properties. Chemical Speciation & Bioavailability.

[CR77] Santoro, N.D. (2020). Lead (Pb) speciation and distribution effects on urban neighborhoods in Akron OH. Kent State University/OhioLINK

[CR78] Schindler M, Hochella MF (2017). Sequestration of Pb–Zn–Sb-and As-bearing incidental nanoparticles by mineral surface coatings and mineralized organic matter in soils. Environmental Science: Processes & Impacts.

[CR79] Schnur J, John RM (2014). Childhood lead poisoning and the new Centers for disease control and prevention guidelines for lead exposure. Journal of the American Association of Nurse Practitioners.

[CR80] Schuch L, Curtis A, Davidson J (2017). Reducing lead exposure risk to vulnerable populations: a proactive geographic solution. Annals of the American Association of Geographers.

[CR81] Schwarz K, Pickett STA, Lathrop RG, Weathers KC, Pouyat RV, Cadenasso ML (2012). The effects of the urban built environment on the spatial distribution of lead in residential soils. Environmental Pollution.

[CR82] Seyferth D (2003). The rise and fall of Tetraethyllead. 2. Organometallics.

[CR83] Smith E, Kempson IM, Juhasz AL, Weber J, Rofe A, Gancarz D, Naidu R, McLaren RG, Gräfe M (2011). In vivo–in vitro and XANES spectroscopy assessments of lead bioavailability in contaminated periurban soils. Environmental Science and Technology.

[CR84] Sowers TD, Bone SE, Noerpel MR, Blackmon MD, Karna RR, Scheckel KG, Juhasz AL, Diamond GL, Thomas DJ, Bradham KD (2021). Plumbojarosite remediation of soil affects lead speciation and elemental interactions in soil and in mice tissues. Environmental Science and Technology.

[CR85] Sperry WA (1948). Paints and protective coatings. Sewage Works Journal.

[CR86] Stewart LR, Farver JR, Gorsevski PV, Miner JG (2014). Spatial prediction of blood lead levels in children in Toledo, OH using fuzzy sets and the site-specific IEUBK model. Applied Geochemistry.

[CR87] Sutherland RA (2003). Lead in grain size fractions of road-deposited sediment. Environmental Pollution.

[CR88] Takaoka M, Yoshinaga J, Tanaka A (2006). Influence of paint chips on lead concentration in the soil of public playgrounds in Tokyo. Journal of Environmental Monitoring.

[CR89] Triantafyllidou S, Edwards M (2012). Lead (Pb) in tap water and in blood: Implications for lead exposure in the United States. Critical Reviews in Environmental Science and Technology.

[CR90] U.S.CensusBureau (2020). https://data.census.gov/profile/Akron_city,_Ohio?g=1600000US3901000

[CR91] USEPA (2023). Reconsideration of the dust-lead hazard standards and dust-lead post-abatement clearance levels (EPA-HQ-OPPT-2023-0231).

[CR92] Wade AM, Richter DD, Craft CB, Bao NY, Heine PR, Osteen MC, Tan KG (2021). Urban-soil pedogenesis drives contrasting legacies of lead from paint and gasoline in city soil. Environmental Science and Technology.

[CR93] Walker SR, Jamieson HE, Rasmussen PE (2011). Application of synchrotron microprobe methods to solid-phase speciation of metals and metalloids in house dust. Environmental Science and Technology.

[CR94] Wang Z, Coyte RM, Cowan EA, Stapleton HM, Dwyer GS, Vengosh A (2021). Evaluation and integration of geochemical indicators for detecting trace levels of coal fly ash in soils. Environmental Science and Technology.

[CR95] Wang Z, Dwyer GS, Coleman DS, Vengosh A (2019). Lead isotopes as a new tracer for detecting coal fly ash in the environment. Environmental Science & Technology Letters.

[CR96] Wang Z, Wade AM, Richter DD, Stapleton HM, Kaste JM, Vengosh A (2022). Legacy of anthropogenic lead in urban soils: Co-occurrence with metal(loids) and fallout radionuclides, isotopic fingerprinting, and in vitro bioaccessibility. Science of the Total Environment.

[CR97] Williams JC, Basu AR, Bhargava ON, Ahluwalia AD, Hannigan RE (2012). Resolving original signatures from a sea of overprint—The geochemistry of the Gungri Shale (Upper Permian, Spiti Valley, India). Chemical Geology.

[CR98] Wu J, Edwards R, He X, Liu Z, Kleinman M (2010). Spatial analysis of bioavailable soil lead concentrations in Los Angeles California. Environmental Research.

[CR99] Yan K, Dong Z, Wijayawardena MAA, Liu Y, Naidu R, Semple K (2017). Measurement of soil lead bioavailability and influence of soil types and properties: A review. Chemosphere.

[CR100] Yeter D, Banks EC, Aschner M (2020). Disparity in risk factor severity for early childhood blood lead among predominantly African-American black children: The 1999 to 2010 US NHANES. International Journal of Environmental Research and Public Health.

[CR101] Zohar I, Bookman R, Levin N, de Stigter H, Teutsch N (2014). Contamination history of lead and other trace metals reconstructed from an urban winter pond in the eastern Mediterranean coast (Israel). Environmental Science and Technology.

[CR102] Zohar I, Teutsch N, Levin N, Mackin G, de Stigter H, Bookman R (2017). Urbanization effects on sediment and trace metals distribution in an urban winter pond (Netanya, Israel). Journal of Soils and Sediments.

