# Supplementary material for: Neighborhood-scale lead (Pb) speciation in Akron, Ohio (USA) soils: primary sources, post-deposition diagenesis, and high concentrations of labile Pb
Source: Environ Geochem Health. 2024 Apr 9;46(5):164. doi: 10.1007/s10653-024-01954-z (PMC11003916; doi:10.1007/s10653-024-01954-z)
Supplement: Supplementary file 2 — Supplementary file2 (DOCX 820 KB) [file 10653_2024_1954_MOESM2_ESM.docx]

Neighborhood-scale lead (Pb) speciation in Akron, Ohio (USA) soils: Primary sources, post-deposition diagenesis, and high concentrations of labile Pb

Nicholas Santoro^1^, David M. Singer^1,^*, Bridget K. Mulvey^2^, Katrina Halasa^3^, Nadya Teutsch^4^, Allie Shedleski^1^, and Madison Wood^1^

1. Department of Geology, Kent State University, Kent, OH 44242, USA

2. School of Teaching, Learning and Curriculum Studies, Kent State University, Kent, OH 44242, USA

3. Akron Public Schools

4. Geochemistry and Environmental Geology Division, Geological Survey of Israel, Jerusalem 9692100, Israel

*corresponding author: dsinger4@kent.edu

*Supporting Information*

**SI Table 1.** Pearson Correlation matrix for total metal concentrations (T) determined by XRF and nitric-acid extractable metal concentrations (AE) determined by ICP-MS for the Akron soil samples (n = 82). The correlation matrix shows positive (blue) and negative (red) correlations, where dark shading represents a strong positive (x > 0.5, dark blue) or negative correlation (x < 0.5, dark red) and light shading (light blue and light red) represent weak correlations (0.5> x <-0.5). White squares fall below a significance level of 0.05.


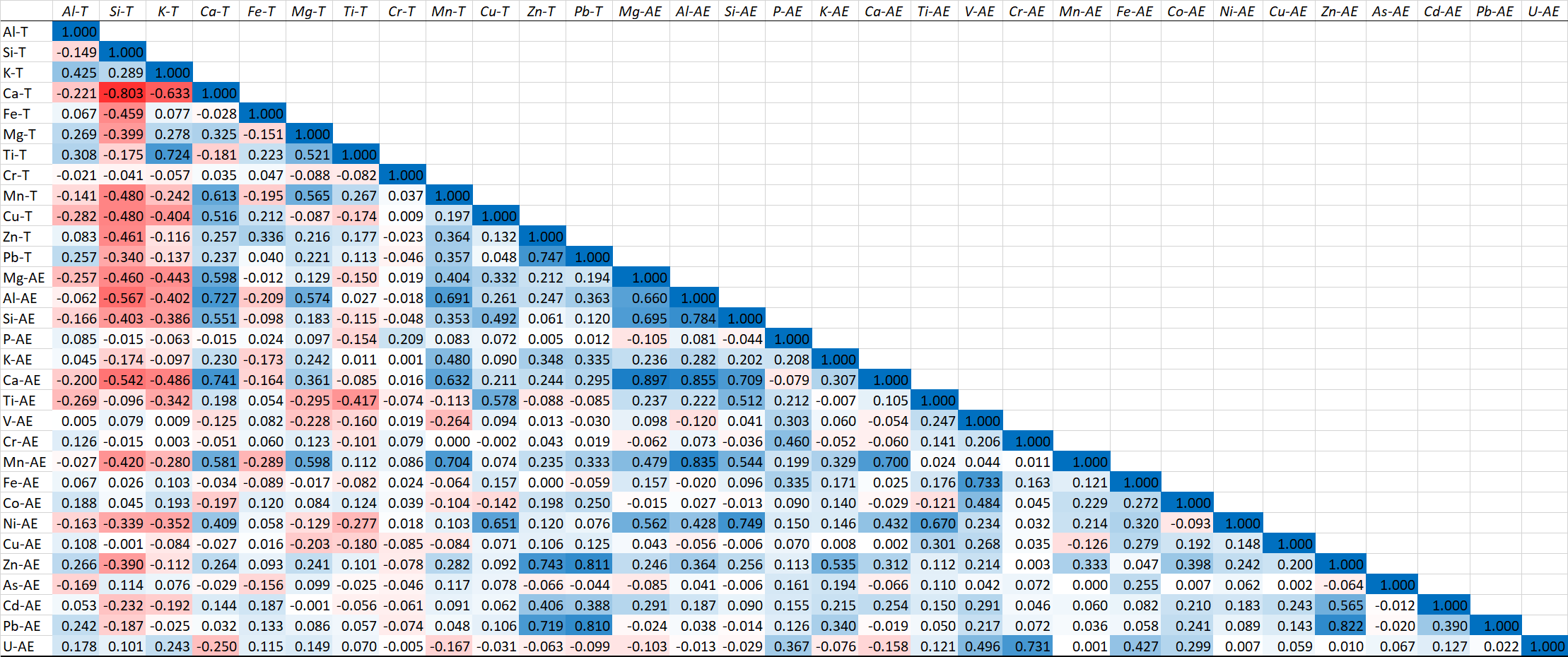

**SI Figure 1**. (A) Percent mineral abundance based on bulk XRD, where feldspars is the sum of anorthite and albite, and phyllosilicates is the sum of kaolinite, muscovite, and biotite (n = 47. (B) Percent loss-on-ignition (n=82).


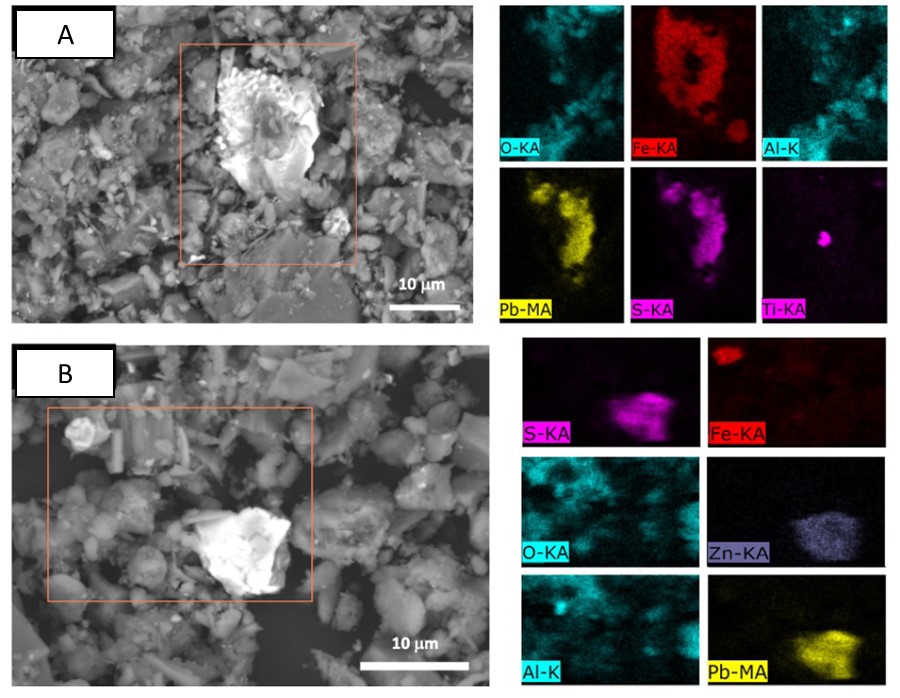


**SI Figure 2.** Additional SEM images and EDS element maps for two samples showing Pb-S-rich phases consistent with the composition and morphology of galena. Both (A) and (B) are from the same sample, which had values for [Pb]_T_ and [Pb]_AE_ of 198 mg/kg and 20 mg/kg, respectively.


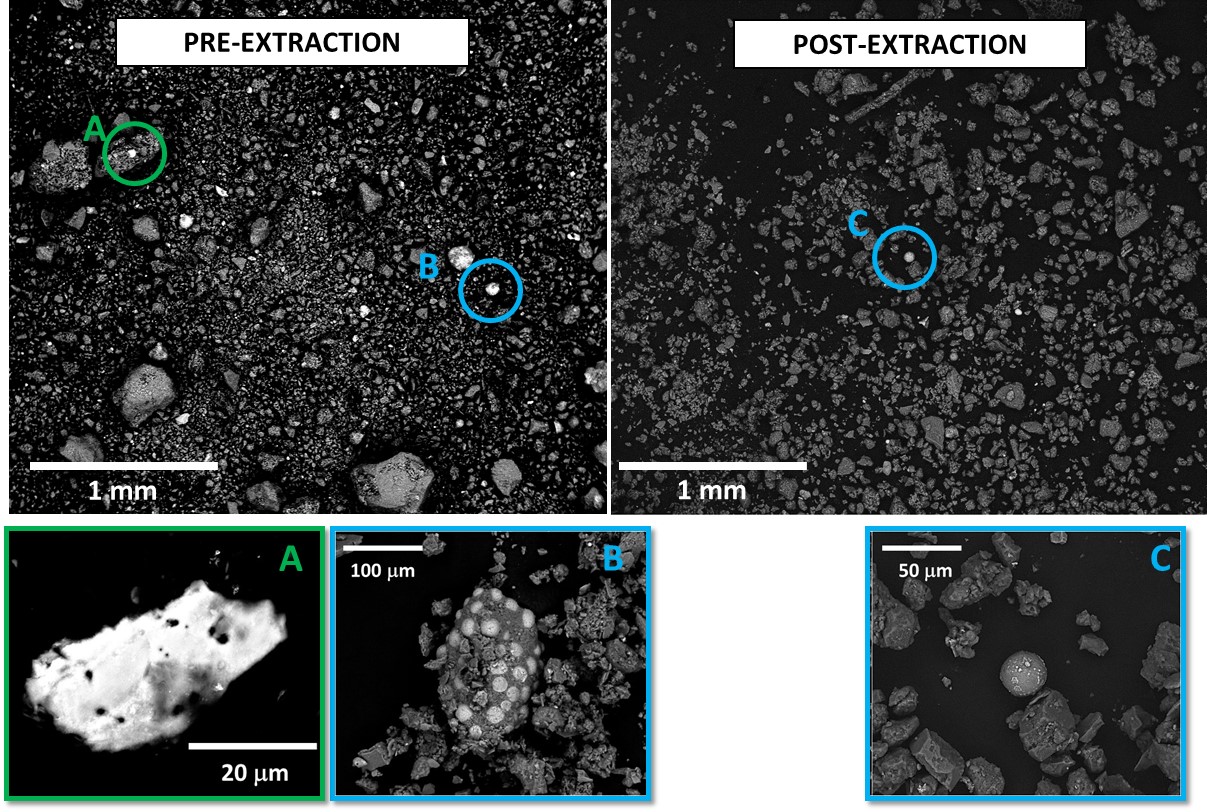


**SI Figure 3.** Representative SEM images of the soil sample shown in Figure 3, with pre- (left) and post-acid extraction (right) highlighting easily identifiable high-contrast metal-rich phases that included discrete Pb-bearing phases (A) and slag (B and C). The presence of discrete Pb-bearing phases could not be identified in post-acid extracted samples.
